# Regulation of the SNARE-interacting protein Munc18c tyrosine phosphorylation in adipocytes by protein-tyrosine phosphatase 1B

**DOI:** 10.1186/1478-811X-11-57

**Published:** 2013-08-12

**Authors:** Jesse Bakke, Ahmed Bettaieb, Naoto Nagata, Kosuke Matsuo, Fawaz G Haj

**Affiliations:** 1 Nutrition Department, University of California Davis, One Shields Ave, 3135 Meyer Hall, Davis, CA 95616, USA; 2 Department of Internal Medicine, University of California Davis, Sacramento, CA 95817, USA; 3 Comprehensive Cancer Center, University of California Davis, Sacramento, CA 95817, USA

**Keywords:** PTP1B, Munc18c, SNARE complex, Adipocytes, Glucose

## Abstract

**Background:**

Protein-tyrosine phosphatase 1B (PTP1B) is a physiological regulator of insulin signaling and adiposity and is a drug target for the treatment of obesity and diabetes. The molecular mechanisms underlying PTP1B metabolic actions require additional investigation.

**Results:**

Herein, we identify Munc18c as a novel PTP1B substrate in adipocytes and *in vivo*. We demonstrate nutritional regulation of Munc18c in adipose tissue revealing decreased expression upon high fat feeding. In addition, PTP1B deficiency leads to elevated Munc18c tyrosine phosphorylation and dissociation from syntaxin4. At the molecular level, we identify Munc18c Tyr^218/219^ and Tyr^521^ as key residues that mediate Munc18c interaction with PTP1B. Further, we uncover an essential role of Munc18c total tyrosine phosphorylation in general, and Tyr^218/219^ and Tyr^521^ in particular, in regulating its interactions and glucose uptake in adipocytes.

**Conclusion:**

In conclusion, our findings identify PTP1B as the first known tyrosine phosphatase for Munc18c and a regulator of its phosphorylation and function in adipocytes.

## Background

Glucose homeostasis is tightly controlled through the regulated balance of absorption from the intestine, production from the liver and uptake by peripheral tissues. Most postprandial glucose uptake in peripheral tissues occurs in muscle and fat. Insulin-stimulated glucose uptake in these tissues is mediated by translocation of vesicles containing the glucose transporter GLUT4 from intracellular stores to the cell periphery and subsequent fusion with the plasma membrane (PM) resulting in the externalization of GLUT4 [1,2]. Fusion of GLUT4 vesicles with the PM is mediated by soluble N-ethylmaleimide-sensitive factor attachment protein receptor (SNARE) complex that consists of synaptosomal-associated protein (SNAP23), syntaxin4 and vesicle-associated membrane protein 2 (VAMP2) [3-5]. SNARE complexes are strictly regulated to ensure that trafficking occurs with precise spatial and temporal coordinates.

Sec1/Munc18 (SM) proteins are essential regulators of SNARE-mediated vesicle budding and fusion events. SM proteins are conserved in *Saccharomyces cerevisiae* (Sec1), *Caenorhabditis elegans* (Unc18) and mammalian systems (Munc18). Mammalian cells express three isoforms (a, b and c) of Munc18 from the SM protein family and are largely localized to the PM [6,7]. Munc18-1 (also known as Munc18a) is expressed predominantly in neuronal tissues and plays an important role in neurotransmitter release [8,9]. Munc18b and Munc18c are ubiquitously expressed [7], though only Munc18c is implicated in the regulation of GLUT4 translocation in adipocytes. 3T3-L1 adipocytes use solely the Munc18c-syntaxin4 pair to regulate insulin-stimulated GLUT4 vesicle exocytosis [10,11]. Studies of Munc18c overexpression in adipocytes or using peptides that inhibit its binding to syntaxin4 reveal an inhibitory role of Munc18c in insulin-stimulated GLUT4 translocation to the PM [11-13]. In line with these findings, adipocytes from Munc18c knockout (KO) mice exhibit increased sensitivity to insulin-stimulated GLUT4 externalization and suggest that Munc18c-syntaxin4 interaction should be disrupted for docking/fusion to proceed [14]. Of note, Munc18c undergoes stimulus-induced tyrosine phosphorylation at Tyr521 to dissociate from syntaxin4 in 3T3-L1 adipocytes [15]. Moreover, the insulin receptor (IR) was identified as a kinase that phosphorylates Munc18c at Tyr521, thereby linking insulin signaling directly to SNARE exocytosis [15,16]. Thus, tyrosine phosphorylation of Munc18c is a regulator of its interactions and function; however, the phosphatase(s) that regulates Munc18c phosphorylation remains unidentified.

Protein-tyrosine phosphatase 1B (PTP1B) is a ubiquitously expressed non-receptor tyrosine-specific phosphatase that is localized on the cytoplasmic face of the endoplasmic reticulum (ER) [17-19]. PTP1B is a physiological regulator of glucose homeostasis and energy balance. Specifically, whole-body PTP1B knockout (KO) mice are hypersensitive to insulin, lean and resistant to high fat diet (HFD)-induced obesity [20,21]. Mice with tissue specific PTP1B deletion in the liver and muscle exhibit improved glucose homeostasis independent of body weight [22-24], while mice with neuronal deletion exhibit decreased body weight [25]. However, the role of PTP1B in adipocytes is unresolved with studies demonstrating detrimental or beneficial effects of adipose PTP1B deficiency on body mass and insulin sensitivity [25,26]. The salutary effects of PTP1B deficiency on obesity and diabetes have focused attention on this phosphatase as a potential therapeutic target. In this study, we identify Munc18c as a novel PTP1B substrate in adipocytes and demonstrate regulation of Munc18c tyrosine phosphorylation and function by PTP1B.

## Results

### PTP1B regulates Munc18c tyrosine phosphorylation

**Figure 1:**
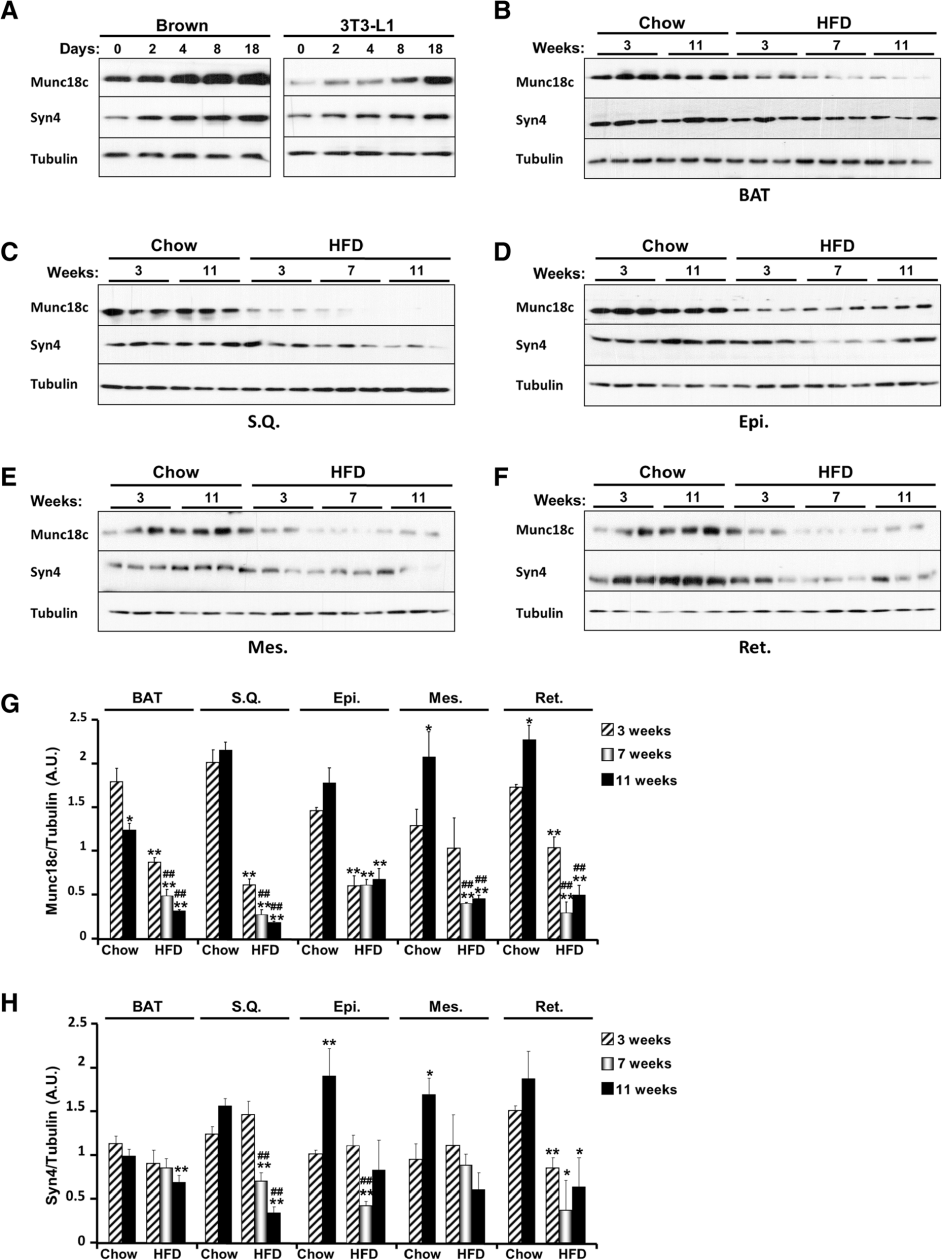
**Munc18c expression in adipocytes and adipose tissue depots. A)** Immunoblots of Munc18c, syntaxin4 and Tubulin in lysates of brown and white (3T3-L1) adipocytes at different stages of differentiation. **B-F)** Immunoblots of Munc18c, syntaxin4 and Tubulin in lysates of brown (BAT), subcutaneous (S.Q.), epididymal (Epi.), mesenchymal (Mes.), and retroperitoneal (Ret.) adipose depots of mice fed regular chow or HFD for the indicated times. Each lane represents a sample from a separate animal. Bar graphs represent normalized data for Munc18c **(G)** and syntaxin4 **(H)** expression normalized to Tubulin and presented as means ± SEM. (*; *P* ≤ 0.05, **; *P* ≤ 0.01) indicate significant difference, in each adipose depot, between all groups versus mice fed regular chow for 3 weeks, and (#; *P* ≤ 0.05, ##; *P* ≤ 0.01) indicate significant difference between mice fed HFD for 7 and 11 weeks versus mice fed HFD for 3 weeks.

The adipose tissue is a regulator of systemic glucose homeostasis and energy balance [27]. There are two major types of adipose tissues in mammals, white and brown. White adipose tissue (WAT) is the main site for triglyceride storage, whereas brown adipose tissue (BAT) plays a role in the defense against cold and has emerging anti-obesity properties [28,29]. To gain insights into the molecular mechanisms underlying PTP1B metabolic actions, we utilized mass spectroscopy and substrate-trapping to determine novel PTP1B substrates in adipocytes [30]. These studies identified known PTP1B substrates indicating the validity of the approach but also uncovered several novel putative substrates including Munc18c. Initially, we examined Munc18c expression in differentiating adipocytes and in adipose tissue depots. Immunoblots of Munc18c in brown [31] and white (3T3-L1) adipose cell lines revealed increased Munc18c expression upon adipocyte differentiation (Figure 1A). In addition, we determined the effect of high fat feeding on Munc18c expression in adipose tissue depots. Mice were fed regular chow diet or HFD and then sacrificed after 3, 7, and 11 weeks. Immunoblots revealed significant decrease in Munc18c expression in mice fed HFD compared with those fed regular chow in all examined adipose depots (Figure 1B-G). Of note, Munc18c cognate syntaxin, syntaxin4 demonstrated comparable expression pattern increasing in adipocytes during differentiation and decreasing in adipose tissue depots upon high fat feeding (Figure 1). These findings demonstrate regulated expression of Munc18c in adipose tissue depots.

**Figure 2:**
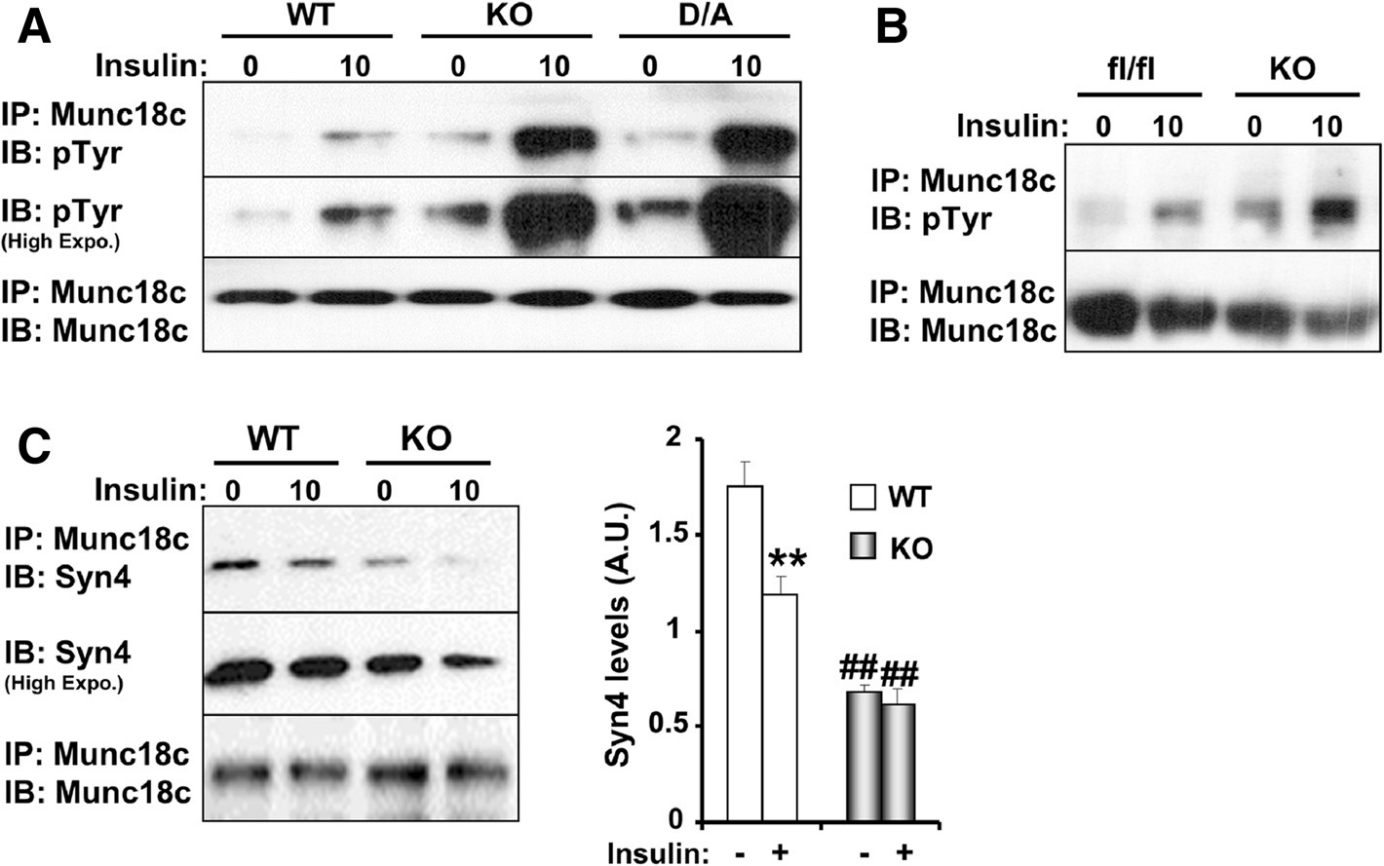
**PTP1B regulates Munc18c tyrosine phosphorylation. A)** Munc18c was immunoprecipitated from lysates of PTP1B KO adipocytes and KO adipocytes reconstituted with PTP1B WT and D/A at basal and insulin-stimulated conditions then immunoblotted using antibodies for phosphotyrosine and Munc18c. **B)** Munc18c was immunoprecipitated from subcutaneous adipose tissue of control (fl/fl) and adipose-specific PTP1B KO fed HFD then immunoblotted using antibodies for phosphotyrosine and Munc18c. **C)** Munc18c was immunoprecipitated from PTP1B KO adipocytes and KO adipocytes reconstituted with WT at basal and insulin-stimulated conditions then immunoblotted using antibodies for syntaxin4 and Munc18c. Bar graph represents normalized data presented as means ± SEM. (**; *P* ≤ 0.01) indicates significant difference between insulin treated and non-treated cells, and (##; *P* ≤ 0.01) indicates significant difference between WT and KO cells for the corresponding treatment.

If Munc18c is a PTP1B substrate then PTP1B deficiency should lead to increased Munc18c tyrosine phopshorylation. To test this, we utilized PTP1B KO adipocytes [31] that are reconstituted with PTP1B wild type (WT) or the substrate-trapping mutant D181A (D/A) that retains substrate binding but is catalytically impaired [32]. Munc18c was immunoprecipitated from starved and insulin-stimulated (10 minutes) KO, WT and D/A adipocytes then immunoblotted using anti-phosphotyrosine antibodies (Figure 2A). In line with previous studies [15,33,34], insulin stimulation increased Munc18c tyrosine phosphorylation in WT adipocytes. Importantly, basal and insulin-stimulated Munc18c tyrosine phosphorylation was significantly elevated in KO and D/A compared with WT adipocytes. To evaluate if adipose PTP1B deficiency *in vivo* modulates Munc18c tyrosine phosphorylation, we determined Munc18c phosphorylation in the subcutaneous adipose depot of control (fl/fl) and adipose-specific PTP1B KO mice. Insulin treatment led to increased Munc18c tyrosine phosphorylation in WT mice compared with basal (Figure 2B). Further, adipose-specific PTP1B KO mice exhibited increased basal and insulin-stimulated Munc18c tyrosine phosphorylation compared with control mice (Figure 2B). Since Munc18c tyrosine phosphorylation disrupts its interaction with syntaxin4 [15,16,33,34], we examined if PTP1B deficiency-induced increase in Munc18c tyrosine phosphorylation results in attenuated interaction with syntaxin4. To that end, Munc18c was immunoprecipitated from starved and insulin-stimulated WT and KO adipocytes then immunoblotted for syntaxin4 (Figure 2C). Indeed, insulin stimulation attenuated Munc18c-syntaxin4 interaction in WT adipocytes compared with basal. Further, basal and insulin-stimulated Munc18c-syntaxin4 co-association was lower in KO compared with WT adipocytes consistent with elevated Munc18c phosphorylation in KO adipocytes. Together, these data demonstrate that PTP1B deficiency in adipocytes and *in vivo* leads to increased Munc18c tyrosine phosphorylation.

### Munc18c is a novel PTP1B substrate

**Figure 3:**
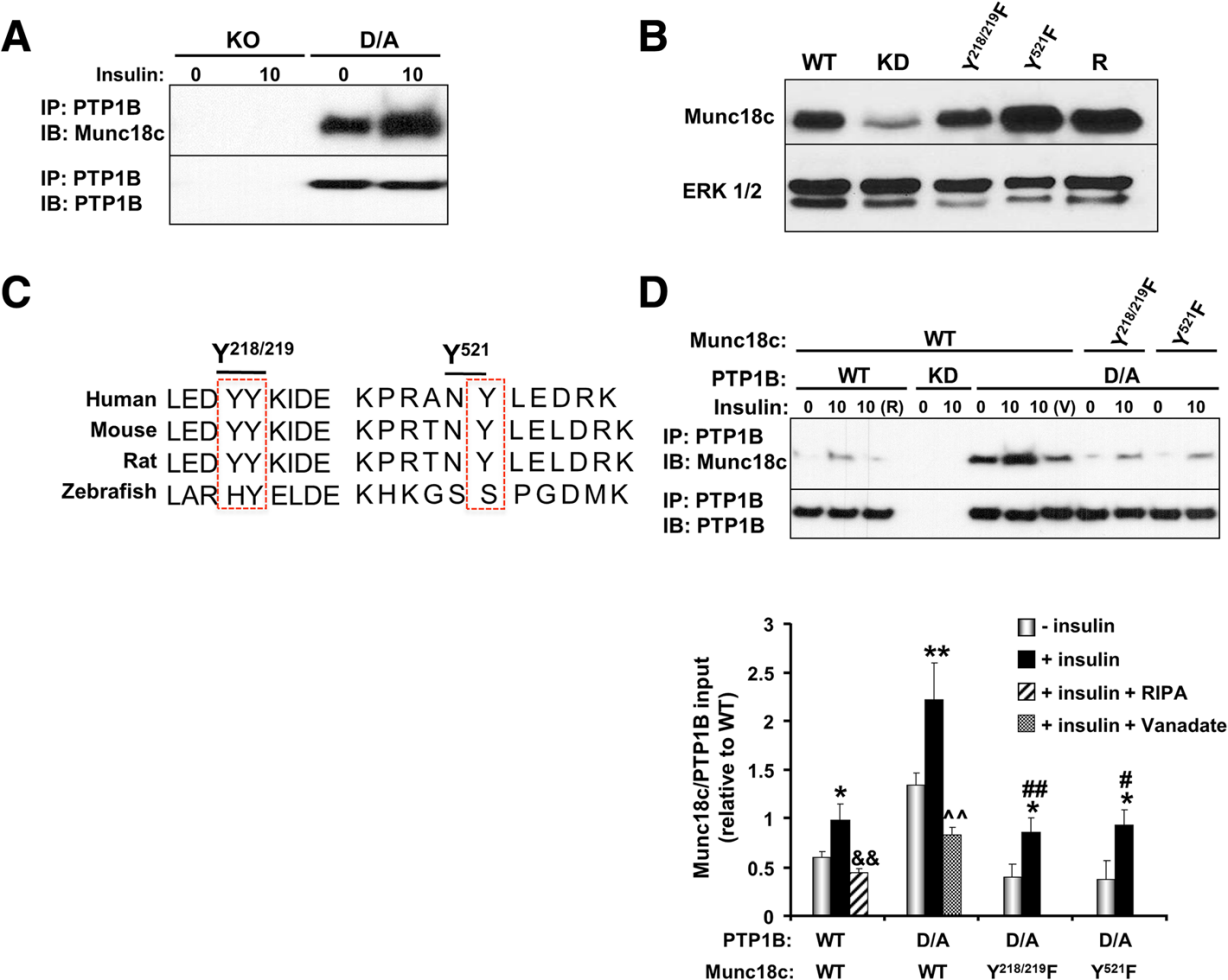
**Munc18c is a PTP1B substrate. A)** PTP1B was immunoprecipitated from lysates of PTP1B KO adipocytes and KO adipocytes reconstituted with PTP1B D/A at basal and insulin-stimulated conditions then immunoblotted using antibodies for Munc18c and PTP1B. **B)** Munc18c knockdown (KD) cells were reconstituted with Munc18c wild type (R) and Y/F mutants (Y^218/219^F and Y^521^F). Lysates were immunoblotted for Munc18c and Erk1/2. **C)** Sequences were aligned relative to amino acids Tyr^218/219^ and Tyr^521^ of mouse Munc18c. The boxed region denotes the location of amino acids Tyr^218/219^ and Tyr^521^ across species. **D)** 3T3-L1 wild type (WT) and Munc18c knockdown cells (reconstituted with Y/F mutants) were transiently transfected with PTP1B WT and D/A. PTP1B was immunoprecipitated using FG6 antibodies from starved and insulin-stimulated cells lysed in NP40 or RIPA (R), resolved using SDS-PAGE then immunoblotted using antibodies for Munc18c and PTP1B. Bar graph represents Munc18c levels in PTP1B immunoprecipitates normalized to level of PTP1B and presented as means ± SEM. (*; *P* ≤ 0.05, **; *P* ≤ 0.01) indicate significant difference between insulin stimulated and non-stimulated cells, (#; *P* ≤ 0.05, ##; *P* ≤ 0.01) indicate significant difference between Munc18c Y/F mutants and Munc18c WT insulin stimulated D/A cells, (&&; *P* ≤ 0.01) indicates significant difference between RIPA-treated and non-treated insulin stimulated WT cells, and (^^; *P* ≤ 0.01) indicates significant difference between vanadate-treated and non-treated insulin stimulated D/A cells.

The elevated Munc18c tyrosine phosphorylation upon PTP1B deficiency promoted us to test whether Munc18c is a direct substrate of PTP1B. Wild type PTP1B has a high catalytic constant rendering its interaction with substrates difficult to detect [35]. However, steady-state interaction of PTP1B with its substrates can be enhanced using the substrate-trapping mutant that forms stable complexes with tyrosine-phosphorylated substrates and has been successfully utilized to identify PTP1B substrates [19,32,36-38]. PTP1B was immunoprecipitated from KO and D/A adipocytes and co-association with Munc18c was determined. As expected, no association was detected in KO adipocytes, but co-association was observed in D/A adipocytes under basal conditions and was significantly enhanced upon insulin stimulation (Figure 3A). To further investigate the mechanism underlying Munc18c-PTP1B interaction, we utilized lentiviral shRNA to generate 3T3-L1 preadipocytes with stable knockdown (KD) of Munc18c. Knockdown cells were reconstituted with wild type Munc18c (R), and tyrosine (Y)-to-phenylalanine (F) mutants Y^218/219^F and Y^521^F (Figure 3B). Munc18c Tyr218/219 and Tyr521 sites were specifically evaluated for several reasons: (**a**) these sites are conserved in many species (Figure 3C), (**b**) both sites are identified biochemically as important for Munc18c-syntaxin4 binding [34], (**c**) Munc18c undergoes stimulus-induced tyrosine phosphorylation at Tyr219 in islet β-cells and Tyr521 in 3T3-L1 adipocytes [15,33,34], (**d**) IR phosphorylates Munc18c at Tyr521 in 3T3-L1 adipocytes [15,16], and (**e**) the amino acid sequence flanking Tyr218/219 is similar to E/D-pY-pY-R/K which has been reported as important for optimal substrate recognition by PTP1B (but is not a requirement) [39]. PTP1B WT and D/A were transiently expressed in WT adipocytes and in adipocytes with Munc18c knockdown reconstituted with Munc18c Y^218/219^F and Y^521^F mutants, then co-association with Munc18c was determined (Figure 3D). We did not expect co-association between PTP1B WT and Munc18c given the transient nature of the interaction. However, modest co-immunoprecipitation was observed upon insulin stimulation (Figure 3D), but is likely indirect and probably due to the presence of both proteins in a large signaling complex. Indeed, lysis of cells using the stringent lysis buffer RIPA (R) disrupted PTP1B WT-Munc18c co-association (Figure 3D, lane 3). As expected, no co-association was observed in cells with PTP1B knockdown (Figure 3D, lanes 4, 5). Consistent with the proposed hypothesis, co-association of endogenous Munc18c and PTP1B D/A was detected under basal conditions and was significantly enhanced upon insulin stimulation (Figure 3D). Importantly, treatment with pervanadate (V), a strong inhibitor of tyrosine phosphatases which oxidizes the essential cysteinyl residue in the catalytic center of the enzymes [40], disrupted Munc18c-PTP1B D/A interaction (Figure 3C, lane 8). This indicated that the association is consistent with enzyme-substrate interaction that is mediated by the active site cysteinyl residue of PTP1B. Of note, Y^218/219^F and Y^521^F mutations significantly attenuated Munc18c association with PTP1B D/A (Figure 3C). Together, these data demonstrate that Munc18c is a direct substrate of PTP1B and that Tyr^218/219^ and Tyr^521^ are mediators of the association.

### Munc18c tyrosine phosphorylation regulates its binding to syntaxin4

**Figure 4:**
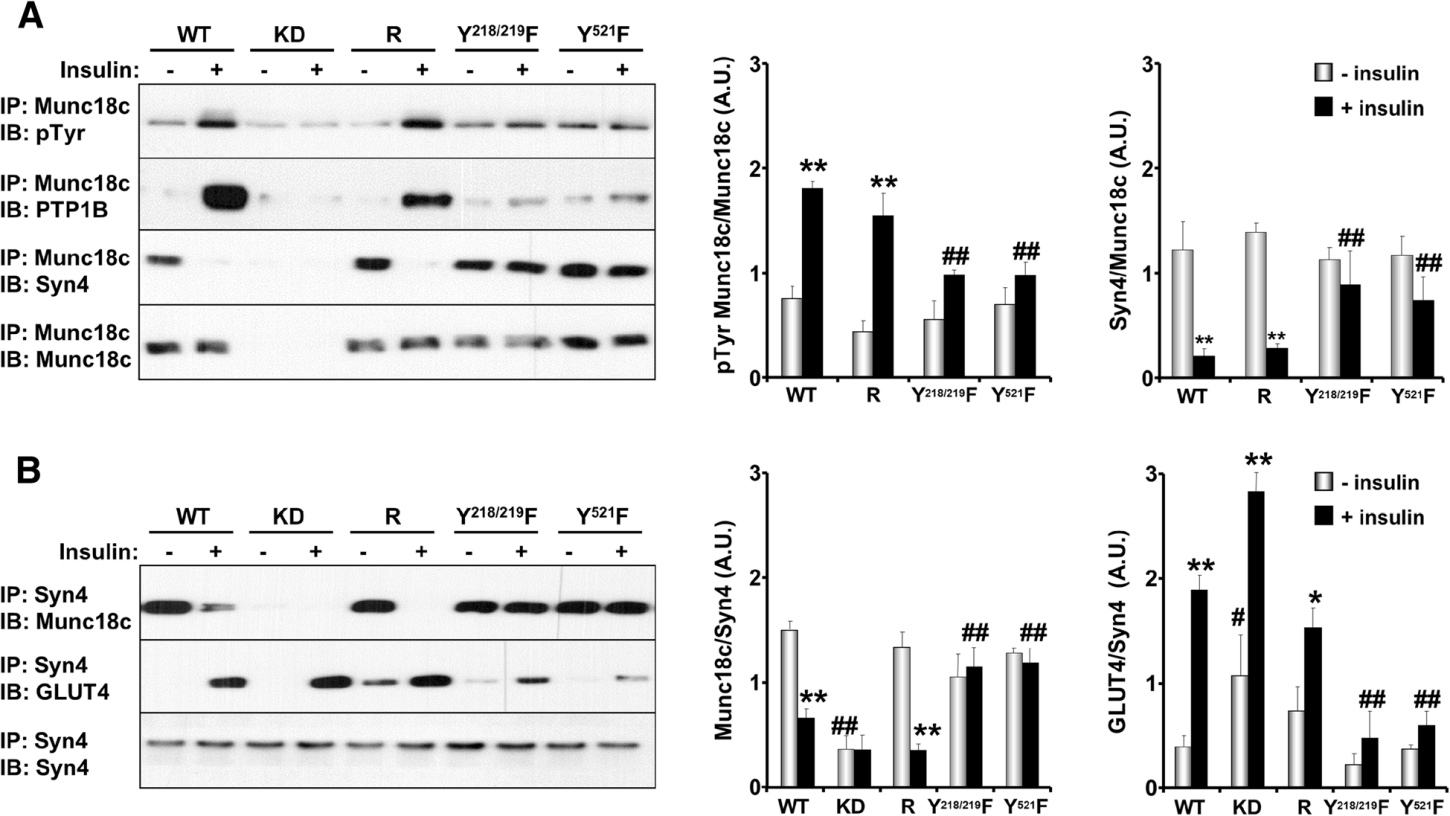
**Tyrosine phosphorylation of Munc18c regulates its binding to syntaxin4 and PTP1B. A)** Munc18c was immunoprecipitated from lysates of wild type adipocytes and adipocytes with knockdown and reconstituted expression of Munc18c (R and Y/F mutants) at basal and insulin-stimulated conditions, then immunoblotted using antibodies for phosphotyrosine, PTP1B, Syn4 and Munc18c. Bar graphs represent normalized data for tyrosine phosphorylated Munc18c/Munc18c and Syn4/Munc18c and presented as means ± SEM. **B)** Syn4 was immunoprecipitated from lysates of WT adipocytes and adipocytes with knockdown and reconstituted expression of Munc18c (R and Y/F mutants) at basal and insulin-stimulated conditions, then immunoblotted using antibodies for Munc18c, Glut4 and Syn4. Bar graphs represent normalized data for Munc18c/Syn4 and Glut4/Syn4 and presented as means ± SEM. (*; *P* ≤ 0.05, **; *P* ≤ 0.01) indicate significant difference between insulin stimulated and non-stimulated cells, and (#; *P* ≤ 0.05, ##; *P* ≤ 0.01) indicate significant difference between different cells versus WT for the corresponding treatment.

**Figure 5:**
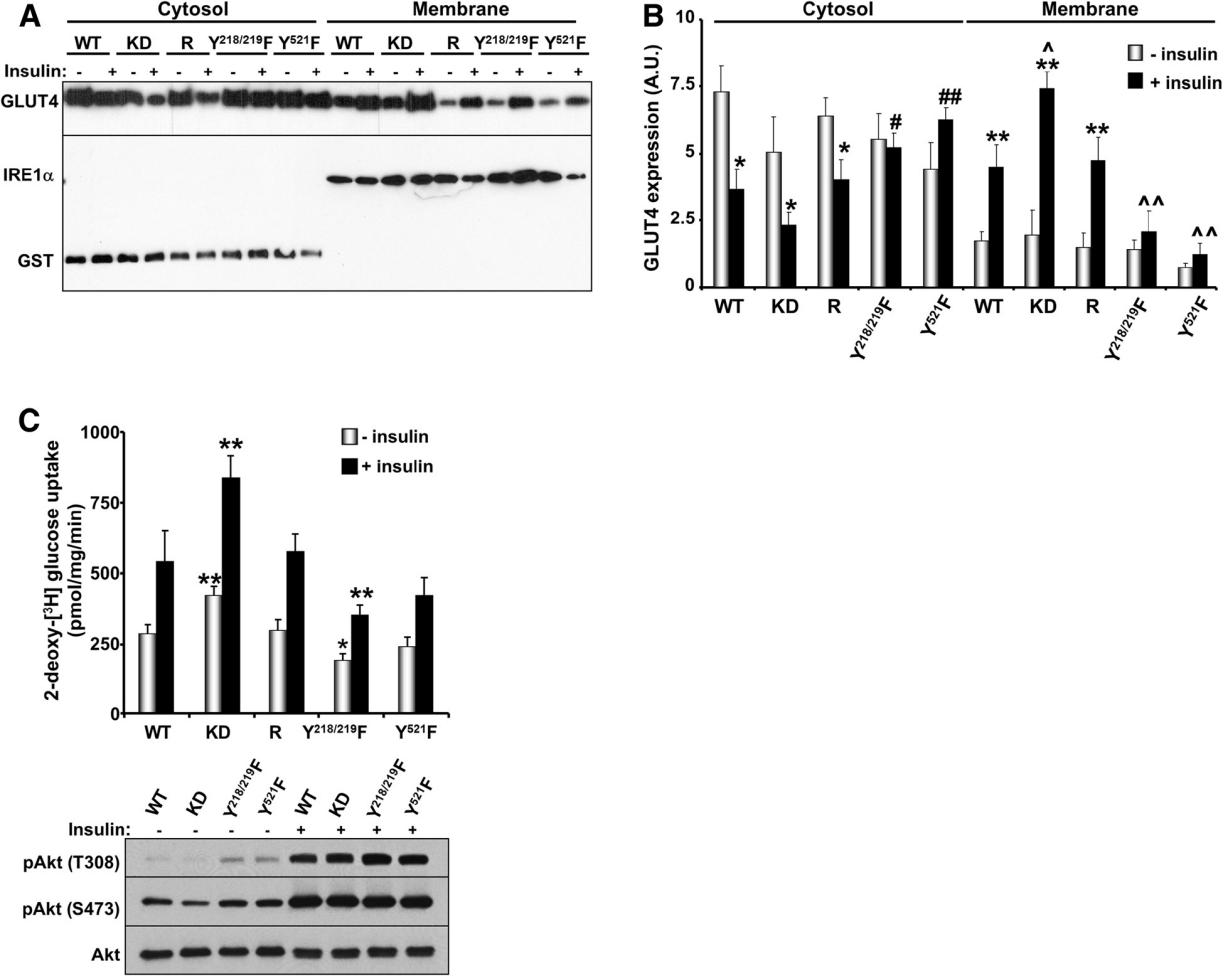
**Tyrosine phosphorylation of Munc18c regulates deoxy-glucose uptake. A)** Immunoblots of GLUT4 in cytosol and membrane fractions of differentiated WT adipocytes and adipocytes with knockdown (KD) and reconstituted expression of Munc18c (R and Y/F mutants) at basal and insulin-stimulated conditions. Gels were also immunoblotted for GST and IRE1α to determine purity of the cytosol and membrane fractions, respectively. **B)** Data are presented as mean ± SEM from four independent experiments. (*; *P* ≤ 0.05, **; *P* ≤ 0.01) indicate significant difference between insulin stimulated and non-stimulated cells in cytosol and membrane fractions, (#; *P* ≤ 0.05, ##; *P* ≤ 0.01) indicate significant difference between different cells versus WT for the corresponding treatement in the cytosol fraction, and (^; *P* ≤ 0.05, ^^; *P* ≤ 0.01) indicate significant difference between different cells versus WT for the corresponding treatment in the membrane fraction. **C)** Basal and insulin-stimulated 2-deoxy-[^3^H] glucose uptake was determined in differentiated WT adipocytes and adipocytes with KD and reconstituted expression of Munc18c at basal and insulin-stimulated conditions. Data are presented as mean ± SEM from five independent experiments. (*; *P* ≤ 0.05, **; *P* ≤ 0.01) indicate significant difference between the different cells versus WT at the corresponding treatment. Immunoblots of pAkt (T308 and S473) and Akt in lysates of differentiated WT adipocytes and adipocytes with KD and reconstituted expression of Munc18c at basal and insulin-stimulated conditions.

We investigated the role of Munc18c total and Tyr^218/219^ and Tyr^521^ phosphorylation in Munc18c interactions and the insulin-stimulated SNARE-mediated delivery of GLUT4 to the plasma membrane. Initially, we determined Munc18c total tyrosine phosphorylation in 3T3-L1 adipocytes (WT) and in adipocytes with knockdown (KD) and reconstituted expression of Munc18c (R, Y^218/219^F and Y^521^F) under basal and insulin-stimulated conditions. Munc18c was immunoprecipitated from NP-40 lysed adipocytes then tyrosine phosphorylation determined using anti-phosphotyrosine antibodies (Figure 4A). WT adipocytes exhibited a low but detectable level of Munc18c tyrosine phosphorylation under basal conditions and this was significantly increased upon insulin stimulation. As expected, Munc18c phosphorylation was attenuated in knockdown cells, while reconstitution with Munc18c (R) restored phosphorylation to control (WT) levels. Of note, Y^218/219^F and Y^521^F adipocytes exhibited impaired insulin-stimulated Munc18c tyrosine phosphorylation (Figure 4A). To further demonstrate regulation of PTP1B/Munc18c co-association by tyrosine phosphorylation, Munc18c immunoprecipitates were immunoblotted for PTP1B. In line with Munc18c tyrosine phosphorylation, insulin significantly enhanced Munc18c co-association with PTP1B and this was significantly attenuated in Y^218/219^F and Y^521^F adipocytes (Figure 4A). Next, we sought to determine the effects of Munc18c phosphorylation on its interactions with syntaxin4, so Munc18c immunoprecipitates were immunoblotted for syntaxin4. WT and reconstituted (R) adipocytes exhibited robust association of syntaxin4 and Munc18c under basal conditions and this was disrupted upon insulin stimulation in line with increased Munc18c tyrosine phosphorylation. Interestingly, whereas syntaxin4 associated with Munc18c under basal conditions in Y^218/219^F and Y^521^F adipocytes, it failed to dissociate upon insulin stimulation (Figure 4A). Similar binding pattern was observed in reciprocal co-immunoprecipitation experiments where syntaxin4 was immunoprecipitated and binding of Munc18c determined (Figure 4B). Importantly, dissociation of Munc18c from syntaxin4 enables binding of GLUT4. Indeed, WT and reconstituted (R) adipocytes exhibited robust insulin-stimulated syntaxin4-GLUT4 association and this was impaired in Y^218/219^F and Y^521^F adipocytes (Figure 4B). It is important to note that syntaxin4-GLUT4 association is in line with deoxyglucose uptake in these adipocytes (see Figure 5C). Together, these findings establish an essential role of tyrosine phosphorylation in general, and Tyr^218/219^ and Tyr^521^ in particular, in regulating Munc18c associations and demonstrate that insulin-induced tyrosine phopshorylation triggers Munc18c dissociation from syntaxin4 thereby facilitating SNARE-mediated delivery of GLUT4 to the plasma membrane.

### Munc18c phosphorylation is required for insulin-stimulated glucose uptake

**Figure 6:**
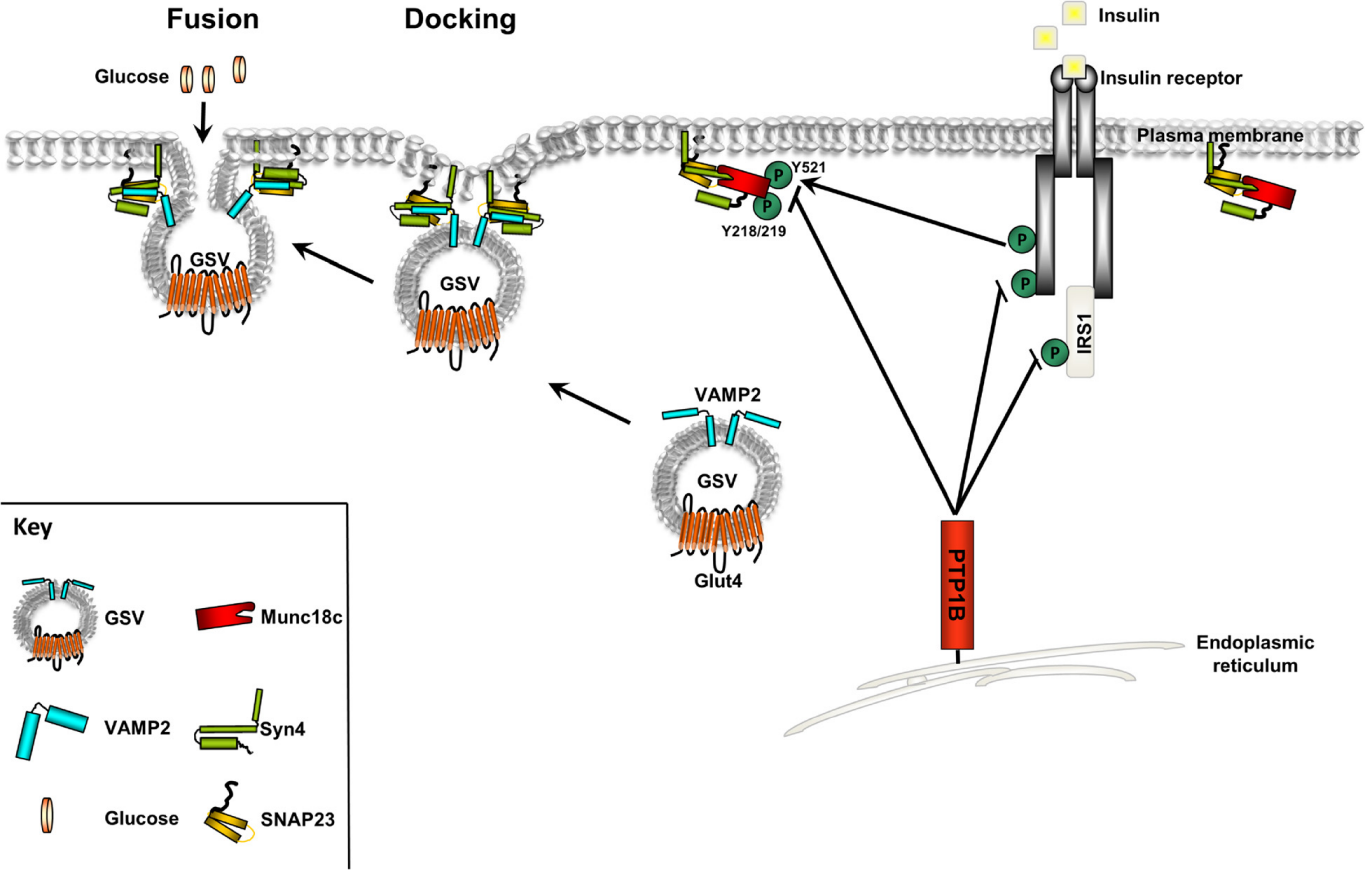
**Simplified schematic representation of GLUT4 traffic to the plasma membrane.** GLUT4 containing vesicles (GSVs) interact with several molecules as they are delivered towards the cell periphery, then tethered, docked and fused to the plasma membrane. The GSV docks with the plasma membrane as a complex is formed between VAMP2 on the vesicle and synatxin4 and SNAP23 on the plasma membrane. This complex then facilitates the fusion of the GSV with the plasma membrane. Tyrosine phosphorylation of Munc18c regulates its binding to syntaxin4. The insulin receptor phosphorylates Munc18c at Tyr^521^ while PTP1B dephosphorylates Munc18c in adipocytes, thereby linking insulin signaling directly to SNARE exocytosis. Adapted from [46] with modifications.

To determine the functional requirements for Munc18c total and site-specific tyrosine phosphorylation in GLUT4 vesicle exocytosis, we evaluated GLUT4 subcellular distribution and 2-deoxy-glucose uptake in differentiated adipocytes. Differentiated Munc18c knockdown and reconstituted adipocytes exhibited comparable triglyceride content (determined using oil Red O staining; data not shown) suggesting that Munc18c does not significantly affect adipogenesis *in vitro*, in line with previous observations [14]. Fractionation of adipocytes revealed increased GLUT4 in membrane fraction of knockdown compared with WT adipocytes (Figure 5A, B). Notably, GLUT4 was significantly decreased in membrane fraction of Y^218/219^F and Y^521^F adipocytes compared with WT (Figure 5A, B). Further, we evaluated basal and insulin-stimulated 2-deoxy-glucose uptake in adipocytes. Insulin stimulation led to clear increase in glucose uptake in control adipocytes (Figure 5C). Importantly, knockdown adipocytes exhibited increased basal and insulin-stimulated glucose uptake compared with WT. This finding is in line with a previous report that demonstrates increased sensitivity to insulin-stimulated GLUT4 externalization in adipocytes of Munc18c knockout mice [14]. In addition, reconstituted (R) adipocytes exhibited comparable basal and insulin-stimulated glucose uptake to WT adipocytes, indicating that the observed effects in knockdown cells are directly due to Munc18c deficiency. On the other hand, basal and insulin-stimulated glucose uptake was attenuated in Y^218/219^F adipocytes and exhibited a trend for attenuation in Y^521^F adipocytes compared with WT (Figure 5C). Finally, Akt activation (determined by phosphorylation at T308 and S473) was comparable between adipocytes. Together, these data demonstrate a requirement for Munc18c tyrosine phosphorylation in insulin-stimulated glucose uptake in adipocytes (Figure 6).

## Discussion

Munc18 proteins are essential regulators of SNARE-mediated vesicle budding and fusion events, and are conserved from yeast to mammals. In this study, we demonstrated nutritional regulation of Munc18c isoform in adipose tissue in mice. In addition, we identified Munc18c as a novel PTP1B substrate in adipocytes and *in vivo* and uncovered Munc18c Tyr^218/219^ and Tyr^521^ as key residues that mediate the association. Further, we established an essential role of Munc18c tyrosine phosphorylation in general, and Tyr^218/219^ and Tyr^521^ in particular, in regulating Munc18c interactions and glucose uptake in adipocytes. Together, these findings identify PTP1B as the first known tyrosine phosphatase for Munc18c and as an important regulator of its phosphorylation and function in adipocytes.

Munc18c expression is regulated in the adipose tissue and chronic high fat feeding in mice leads to decreased Munc18c protein. Expression of the cognate syntaxin, syntaxin4 is also decreased in adipose tissue upon high fat feeding consistent with tight control of Munc18c/syntaxin4 interaction and coordinate regulation of these proteins. The current findings are in line with a recent report demonstrating down regulation of Munc18c protein expression in visceral and subcutaneous adipose tissue depots of morbidly obese human subjects [41]. It is not clear if decreased Munc18c expression in overweight/obese rodents and humans is directly attributed to obesity or other factor(s) such as elevated proinflammatory response to HFD. We speculate that down regulation of Munc18c presents a mode of regulation that may serve as a compensatory mechanism to facilitate GLUT4 translocation and modulate insulin action. Indeed, Munc18c expression decreases after prolonged fasting in skeletal muscle of lean and obese subjects concurrently with reduced insulin action [42]. Understanding the different tiers of Munc18c regulation, such as expression and tyrosine phosphorylation as presented herein, will yield insights into GLUT4 trafficking and possible modalities to increase glucose transport.

Munc18c is a novel PTP1B substrate as evidenced by several supporting observations. Mass spectrometry of adipocytes expressing the substrate-trapping PTP1B mutant identified Munc18c as a PTP1B-interacting protein. In addition, adipocytes and adipose tissue lacking PTP1B exhibited increased Munc18c tyrosine phosphorylation. Moreover, substrate trapping demonstrated direct association between Munc18c and PTP1B D/A. Notably, pervanadate treatment disrupted Munc18c-PTP1B D/A association indicating that it is consistent with direct enzyme-substrate interaction that is mediated by the PTP1B active site. At the molecular level we identified Munc18c Tyr^218/219^ and Tyr^521^ as key residues that mediate the association with PTP1B. It is important to note that structurally these tyrosine residues are predicted to be in close proximity [16]. In addition, Tyr^521^ is in the disordered region of Munc18c that is notable for posttranslational modifications and conformation changes [16]. Importantly, Tyr^521^ was recently identified as the residue that is phosphorylated by IR upon insulin stimulation [15,16]. Therefore, Munc18c tyrosine phosphorylation is regulated, at least in large part, by the opposing actions of IR and PTP1B with precise spatial and temporal coordinates. In one scenario, insulin stimulation leads to IR activation and phosphorylation of Munc18c Tyr^521^ allowing for signal propagation that is then followed by dephosphorylation by PTP1B (Figure 6). Indeed, insulin stimulation leads to reversible oxidation of PTP1B at its active site and transient attenuation of its enzymatic activity [43]. One function of insulin-induced oxidation is transient inactivation of PTP1B, which usually exerts inhibitory constraint on the system, to initiate a signaling response to the stimulus. It is important to note that additional protein-tyrosine phosphatase(s) may also regulate Munc18c phosphorylation. Further, Munc18c is expressed in additional metabolic cells/tissues such as pancreatic β-cells where PTP1B is also expressed [44], and additional studies are required to determine if PTP1B regulates Munc18c (or other isoforms) in these cells. At any rate, the current findings are the first to identify a protein-tyrosine phosphatase that directly regulates Munc18c tyrosine phosphorylation and function in adipocytes.

The current findings demonstrate that Munc18c tyrosine phosphorylation regulates its interactions and are consistent with a functional requirement for Munc18c phosphorylation in glucose uptake in adipocytes. Munc18c tyrosine phosphorylation was elevated upon PTP1B deficiency (in adipocytes and *in vivo*) leading to concomitant decrease (~ 60%) in Munc18c-syntaxin4 association. Comparable attenuation of Munc18c-syntaxin4 association was reported in adipocytes upon insulin stimulation [33] or pervanadate treatment (i.e. PTP inhibition) [16]. In particular, mutants of the tyrosine residues (Tyr^218/219^ and Tyr^521^) that mediate Munc18c-PTP1B interaction lacked the ability to dissociate from syntaxin4 upon insulin stimulation (Figure 4B) demonstrating the importance of these tyrosines in regulating Munc18c interactions. Importantly, we demonstrated that Munc18c association with syntaxin4 is of functional significance. Munc18c deficiency facilitated syntaxin4-GLUT4 interaction leading to enhanced glucose uptake. On the other hand, Munc18c mutants that did not dissociate from syntaxin4 disrupted its binding to GLUT4 and consequently attenuated glucose uptake. Together, our findings demonstrate an important role for PTP1B in regulating Munc18c tyrosine phosphorylation and function. Presumably PTP1B can engage different substrate(s) in adipocytes depending on the nature of the stimulus, its magnitude and duration. Given the salutary effects of PTP1B deficiency and inhibition in obesity and diabetes additional studies are warranted to fully elucidate the substrates and signaling mechanism underlying its metabolic actions.

## Methods

### Reagents

Dulbecco’s Modified Eagle Medium (DMEM), G418, penicillin/streptomycin, puromycin, new born calf serum (NBCS), fetal bovine serum (FBS) and trypsin were purchased from Invitrogen (Carlsbad, CA). Antibodies for human PTP1B (FG6), mouse PTP1B, and Tubulin were from Abcam (Cambridge, MA). Akt, phosphotyrosine (PY99), syntaxin4, Glutathione-S-Transferase pi 1 (GST-π1), IRE1α, and GLUT4 antibodies were from Santa Cruz Biotechnology (Santa Cruz, CA). pAkt (Ser473) and pAkt (Thr308) were from Cell Signaling Technology (Beverly, MA). Munc18c antibodies were from Sigma (St. Louis, MO) and phosphotyrosine (4G10) antibodies were from Millipore (Billerica, MA). Horseradish peroxidase (HRP)-conjugated secondary antibodies were from BioResources International (Carlsbad, CA). Unless otherwise indicated, chemicals were purchased from Sigma.

### Mouse studies

Male mice on C57Bl/6 J background (Jackson Laboratories) were maintained on a 12-hour light–dark cycle and fed *ad libitum*. Mice were fed standard laboratory chow (Purina lab chow, # 5001) at weaning or switched to a HFD (60% kcal from fat, # D12492, Research Diets) at 7 weeks of age. PTP1B-floxed (PTP1B^fl/fl^) mice were generated previously [25]. Adiponectin (Adipoq)-Cre mice were obtained from Dr. E. Rosen (BIDMC/Harvard University). Genotyping for the PTP1B floxed allele and presence of Cre was performed by polymerase chain reaction (PCR), using DNA extracted from tails [25,38]. At the indicated age, mice were sacrificed and adipose depots collected, frozen in liquid nitrogen then stored at −80°C. Mouse studies were approved by Institutional Animal Care and Use Committee at University of California Davis.

### Cell culture

3T3-L1 cells were maintained in DMEM containing 25 mM glucose, 10% bovine calf serum (BCS), 50 U/ml penicillin and 50 μg/ml streptomycin. Munc18c was silenced in 3T3-L1 cells by testing five different hairpins (Open Biosystems). Packaging (psPAX2) and envelope (pMD2.G) vectors (Addgene, Boston) were co-transfected, along with silencing hairpins into HEK293FT cells using Lipofectamine 2000 (Invitrogen) following manufacturer’s guidelines. Lentivirus was collected and used to infect 3T3-L1 cells. Cells were selected using puromycin (2 μg/ml) and drug-resistant pools propagated. Cells with knockdown (KD) of Munc18c were reconstituted by transfection of wild type (WT) mouse Munc18c (Origene) and Y/F (Y^218/219^F and Y^521^F) mutants. Stable cell lines were maintained under 400 μg/ml G418 antibiotic selection for three weeks. To induce cell differentiation, 3T3-L1 preadipocytes were grown to confluence in culture medium containing 10% NBCS. Confluent cells were then cultured in differentiation media containing 10% FBS, 20 nM insulin and 1 nM triiodothyronine [T3] for 48 h. Adipocyte differentiation was induced by treating cells for 48 h in differentiation medium further supplemented with 0.5 μM dexamethasone, 0.5 mM isobutylmethylxanthine, and 0.125 mM indomethacin (induction media). After induction, cells were cultured in differentiation medium until they exhibited a differentiated phenotype with massive accumulation of fat droplets.

### Biochemical analyses

Tissues and cells were lysed in radio-immunoprecipitation assay buffer (RIPA: 10 mM Tris–HCl, pH 7.4, 150 mM NaCl, 0.1% sodium dodecyl sulfate [SDS], 1% Triton X-100, 1% sodium deoxycholate, 5 mM EDTA, 1 mM NaF, 1 mM sodium orthovanadate and protease inhibitors). Lysates were clarified by centrifugation at 13,000 rpm for 10 min, and protein concentrations were determined using a bicinchoninic acid assay kit (Pierce Chemical). For substrate-trapping experiments, cells were lysed in 1% NP40 buffer with a protease inhibitor cocktail (without sodium orthovanadate). Proteins (500 μg) were immunoprecipitated with Munc18c or FG6 antibodies and immune complexes were collected on protein G-Sepharose beads (GE Healthcare) and washed with lysis buffer. Proteins were resolved by SDS-PAGE and transferred to PVDF membranes. Immunoblots were performed using phosphotyrosine (4G10/PY99), FG6, and Munc18c antibodies. Proteins were detected using enhanced chemiluminescence (Amersham Biosciences). Resulting immunoreactive bands were quantified using FluorChem Q Imaging software (Alpha Innotech).

For site-directed mutagenesis, tyrosine (Y) to phenylalanine (F) mutations in mouse Munc18c cDNA (origene) were generated using a QuikChange Lightning Site-Directed Mutagenesis Kit (Stratagene, La Jolla, CA) following the manufacturer instructions and confirmed by DNA sequence analysis.

### Subcellular fractionation

Subcellular fractions were isolated as previously described [45] with modifications. Briefly, differentiated adipocytes were washed in buffer 1 (100 mM sucrose, 1 mM EGTA, 20 mM MOPS, pH 7.4) and resuspended in buffer 2 (100 mM sucrose, 1 mM EGTA, 20 mM MOPS, 1% Triton X-100, 5% Percoll, 0.01% digitonin, 2 mM sodium orthovanadate, 10 mM aprotinin, 10 mM pepstatin A, 10 mM leupeptin, 25 mM calpain inhibitor I and 1 mM PMSF, pH 7.4). Cells were passed through a 30-gauge syringe and incubated for 15 min on ice, unbroken cells, mitochondria and nuclei were pelleted by centrifugation at 15,000 g. The supernatant was centrifuged at 100,000 g for 1 h. The resulting supernatant and pellet were designated as the cytosol and membrane fractions, respectively and membrane fractions were resuspended in RIPA buffer.

### Deoxy-glucose uptake assay

Glucose uptake was determined as previously described [31]. Briefly, differentiated 3T3-L1 adipocytes were treated with insulin for 30 minutes after which 2-deoxy-[^3^H] glucose (0.5 μCi/ml, final concentration) was added for additional 3 minutes. The incorporated radioactivity was quantitated using liquid scintillation counting.

### Statistical analyses

Data are expressed as means ± standard error of the mean (SEM). Statistical analyses were performed using JMP program (SAS Institute). Comparisons between groups were performed using unpaired two-tailed Student’s *t*-test. A symbol (such as *) indicates *P* ≤ 0.05, whereas a duplicate symbol (such as **) indicates *P* ≤ 0.01.

## Abbreviations

PTP1B: Protein-tyrosine phosphatase 1B; Munc18c: Mammalian homolog of Unc-18c; SNARE: Soluble N-ethylmaleimide-sensitive factor attachment protein receptor; SNAP32: Synaptosomal-associated protein; VAMP2: Vesicle-associated membrane protein 2.

## Competing interests

The authors declared that they have no competing interests.

## Authors’ contributions

JB: performed research and analyzed data. AB: designed and performed research, analyzed data and revised the manuscript. NN: performed research and revised the manuscript. KM: performed research. FGH: designed research, analyzed data and wrote the manuscript. All authors read and approved the final manuscript.
